# Supplementary material for: Multimodal false information detection method based on Text-CNN and SE module
Source: PLoS One. 2022 Nov 23;17(11):e0277463. doi: 10.1371/journal.pone.0277463 (PMC9683577; doi:10.1371/journal.pone.0277463)
Supplement: Attachment — Submitted filename: Response to Reviewer #2.pdf [file pone.0277463.s001.pdf]

Response to Reviewer #2

Dear Editors and Reviewers,

On behalf of my co-authors, i am sincerely grateful to you for giving us the opportunity to revise our manuscript entitled “Multimodal false information detection method based on Text-CNN and SE module” (ID: PONE-D-22-26288). We sincerely thank you again for thoroughly examining our manuscript and providing very positive and constructive comments and suggestions to guide our revision.

We have carefully studied your comments and suggestions. In accordance with these comments and suggestions, the manuscript was revised to the best of our ability. Our responses and revisions to each comment and suggestion are shown below. We sincerely hope that this revised manuscript has addressed all your comments and suggestions.

Thank you again for giving your valuable time to review my manuscript. The main corrections in our manuscript and the responds to the reviewers’ comments are as following:

Point 1: Kindly elaborate about your proposed work along with Deep Learning techniques / algorithms in detail.

Response 1: Thank you for your suggestion.

First, we describe our pre-processing process for the data. We resize all the images to (224, 224, 3) and fix the text length (33 for English data and 95 for Chinese data) to be processed in the form of '[CLS]+Text+[SEP]'.

We use the pre-processed data to fine-tune the two pre-trained models, Swin-Transformer and BERT, to make these two models perform better in our task.

Finally, we will introduce our proposed model in detail. Our model first inputs the image with the size of (224,224, 3) into the fine tuned Swin Transformer to obtain the image feature with the size of (49,768), and then inputs the preprocessed text into the fine tuned BERT to obtain the text feature with the size of (33/95,768). Then the image features and text features are processed by (1,768), (2,768) and (3,768) convolution kernels to obtain three (1,64) image features and three (1,64) text features. At the same time, we fuse (49,768) dimensional image features and (33/95,768) dimensional text features to obtain (1, 49) image features and (1, 33/95) text features through our proposed MSE module. Finally, we concatenate three (1,64) and one (1,49) dimensional image features with three (1,64) and one (1,33/95) text features to obtain a joint feature (1,466/528). Finally, we input the (1,466/528) dimensional joint features into a full connection layer with the activation function softmax to obtain the final classification results.

Point 2: In the dataset the text information contains Chinese language, how you will identify the false news?

Response 2: Thank you for your suggestion.

BERT is a powerful text pre-training model that can handle not only English text, but also Chinese text. So for Chinese text we will use the same processing method as English text to discriminate false information, the only difference is the inconsistent length of the text. For the English text, we

keep the text length to 33, while the Chinese text length is kept to 95.

Point 3: Refer to the existing model technique, Kindly compare with your proposed work. Detail explanation is required about your implementation work. (Tools, language, flow chart, etc).

Response 3: Thank you for your suggestion. This was an oversight on our part.

Machine configuration and environment for this experiment: CPU: Intel Xeon E5-2630L v3, 62 G memory, 8cores, GPU: NVIDIA GeForce RTX 3090, PyTorch(1.7.1), Python(3.8), Cuda(10.2)

Point 4: Twitter dataset analyses and implementation details required. (Webio dataset implementation adequate details provided in manuscript).

Response 4: Thank you very much for your valuable comments.

We introduced how to process the Twitter dataset in "Dataset and experimental settings".As to why we do not analyze the Twitter dataset separately, there are several reasons :

1.For the Twitter dataset, the processing is done in a similar way to the Weibo dataset except for the inconsistent text length.

2.The Weibo dataset is more representative than the Twitter dataset. In terms of data volume, the Weibo dataset has more than 30 times more image data than the Twitter dataset. In terms of data composition, the Weibo dataset is closer to real life because most of the posts in the Weiboi dataset are from different events, while most of the events in the Twitter dataset are from the same event. Therefore, we choose the more representative Weibo dataset to do the ablation experiment.

3.Repeating our ablation experiments on the Twitter dataset is not necessary. We set up multiple sets of ablation experiments to validate the effectiveness of our proposed model. To ensure the objectivity of the experimental results, we choose to do all the ablation experiments on the more representative Weibo dataset. The effectiveness of our model has been validated on the more complex Weibo dataset, and we do not consider it necessary to repeat the above ablation experiments on the simpler Twitter dataset.

Point 5: Add more details about Squeeze-and-Excitation Networks.

Response 4: Thank you very much for your valuable comments. We add more details about Squeeze-and-Excitation Networks in the 'Feature fusion' section.The added content is shown below.

The SE module is mostly used for channel feature enhancement of the input feature maps in computer vision tasks. For example, if we input a feature map A with dimensions (H,W,C), the SE module will input A into two full connection layers to get the attention score, and then multiply the feature map A with the attention score in the dimension of the channel to get the final output.

Point 6: Kindly mention the total dataset used in each model in table no.3 (Include one more column).

Response 4: Thank you very much for your valuable comments.

We mention the datasets used by each model in Table 3. The first column of Table 3 shows the dataset used by the model. As shown in Table 3, the Twitter dataset and the Weibo dataset are followed by the experimental results of seven different models on that dataset.

Thank the reviewer for the kind advice again. If you have any questions, please contact us without hesitation.

Yours sincerely
